# The significance of epigenetic alterations in lung carcinogenesis

**DOI:** 10.1007/s11033-012-2063-4

**Published:** 2012-10-20

**Authors:** Ewa Brzeziańska, Agata Dutkowska, Adam Antczak

**Affiliations:** 1 Department of Molecular Bases of Medicine, Medical University of Lodz, Pomorska St. 251, 92-213 Lodz, Poland; 2 Department of Pneumology and Allergology, Medical University of Lodz, Kopcinskiego St. 22, Lodz, Poland

**Keywords:** Epigenetic modifications, Promoter hypermethylation, Histone modifications, miRNA, Lung cancer

## Abstract

Lung cancer is recognized as a leading cause of cancer-related death worldwide and its frequency is still increasing. The prognosis in lung cancer is poor and limited by the difficulties of diagnosis at early stage of disease, when it is amenable to surgery treatment. Therefore, the advance in identification of lung cancer genetic and epigenetic markers with diagnostic and/or prognostic values becomes an important tool for future molecular oncology and personalized therapy. As in case of other tumors, aberrant epigenetic landscape has been documented also in lung cancer, both at early and late stage of carcinogenesis. Hypermethylation of specific genes, mainly tumor suppressor genes, as well as hypomethylation of oncogenes and retrotransposons, associated with histopathological subtypes of lung cancer, has been found. Epigenetic aberrations of histone proteins and, especially, the lower global levels of histone modifications have been associated with poorer clinical outcome in lung cancer. The recently discovered role of epigenetic modifications of microRNA expression in tumors has been also proven in lung carcinogenesis. The identified epigenetic events in lung cancer contribute to its specific epigenotype and correlated phenotypic features. So far, some of them have been suggested to be cancer biomarkers for early detection, disease monitoring, prognosis, and risk assessment. As epigenetic aberrations are reversible, their correction has emerged as a promising therapeutic target.

## Introduction

Nowadays it is clearly acknowledged, that during neoplastic transformation genetic lesions are accompanied by equally important epigenetic modifications. The altered methylation pattern of promoter sequence can cause the same effect as the mutation in coding region of the gene and may be subject to the same processes of selection as genetic changes during carcinogenesis [1, 2]. What’s more, the mutual interaction between the two mechanisms—genetic and epigenetic—has been confirmed [3–5]. Epigenetic modifications may lead to point mutations, as in case of epigenetically silenced DNA repair genes, and conversely, genetic mutations can disturb normal methylation pattern [6, 7]. Additionally, it has been observed that epigenetic modifications are much more diverse and frequent than DNA mutations [1, 3, 8].

Epigenetic mechanisms, related to inherited changes in gene expression without alterations in the primary DNA sequence [8, 9], are essential for normal cellular functioning. They play particularly important role in genome activity regulations, encompassing key biological processes, such as differentiation and embryonic development, tissue-specific gene expression regulation or imprinting and X chromosome silencing in females. Epigenetic mechanisms comprise DNA methylation, histone modifications, nucleosome positioning, and small, noncoding RNAs (miRNA, siRNA) regulation. During the last few decades, epigenetic alterations are widely described as essential players in cancer development and progression [10]. Epigenetic changes have been identified as putative cancer biomarkers for early detection, cancer management and monitoring, as well as disease prognosis, and risk assessment.

As so far, the best known epigenetic modifications observed in human tumor cells are aberrant DNA methylation patterns—the first epigenetic alterations identified in cancer—and nucleosomal histone modifications. The first articles on this subject were published in 1980s. The subsequent studies showed that the spatial conformation of chromatin, associated with nucleosome positioning, also affects gene expression. Recently, the role of non-coding RNA (ncRNA) in the epigenetic control has been described. Publications regarding this level of epigenetic signature have appeared during the last 4 years. So, the molecular mechanisms underlying epigenetic regulatory processes have been discovered relatively recently, and the knowledge of this field is still enriched. The most frequent epigenetic modifications in cancer cell and the involved mechanisms are summarized in Table 1.

**Table 1:** The examples of epigenetic modifications and the underlying molecular mechanisms in cancer cell [8, 147]

Epigenetic modification and their molecular mechanisms
DNA methylation
Hypermethylation: covalent binding of methyl groups (-CH_3_) to cytosines within CpG dinucleotide-rich DNA sequence (CpG islands) Hypomethylation: separation of methyl groups from normally methylated cytosines in DNA sequence
Histone modifications
Methylation, acetylation, phosphorylation, ubiquitination, glycosylation, ADP-ribosylation, and sumoylation of amino acid residues (K/Lys/, S/Ser/, T/Thr/, R/Arg/) in core histones
Nucleosome positioning
ATP-dependent chromatin remodeling complexes and nucleosomal remodeling factors (NuRFs) activity; histone variant (H3.3, H2A.Z) binding to gene promoter
Non-coding RNA (ncRNAs) epigenetic deregulations
Post-transcriptional gene expression regulation via miRNA binding with RNA-induced silencing complex (RISC) possessing endoribonuclease activity (RNA interference), epigenetic silencing of miRNA genes (promoter hypermethylation/hypomethylation; histone modifications); DNMT targeting

The final result of epigenetic modifications, listed in Table 1, is the altered structure of chromatin involved in packaging of the human genome. This process, the so called chromatin remodeling, is associated with chromatin conformational changes into functionally active structure (euchromatin) or inactive condensed form (heterochromatin) [1, 11, 12]. The additional epigenetic level in gene expression control is carried out by deregulation of non-coding RNA function and post-transcriptional miRNA control of 3′untranslated regions (3′UTR) of target messenger RNAs via transcript destabilization and mRNAs repression and/or inhibition of translation [13].

**Fig. 1:**
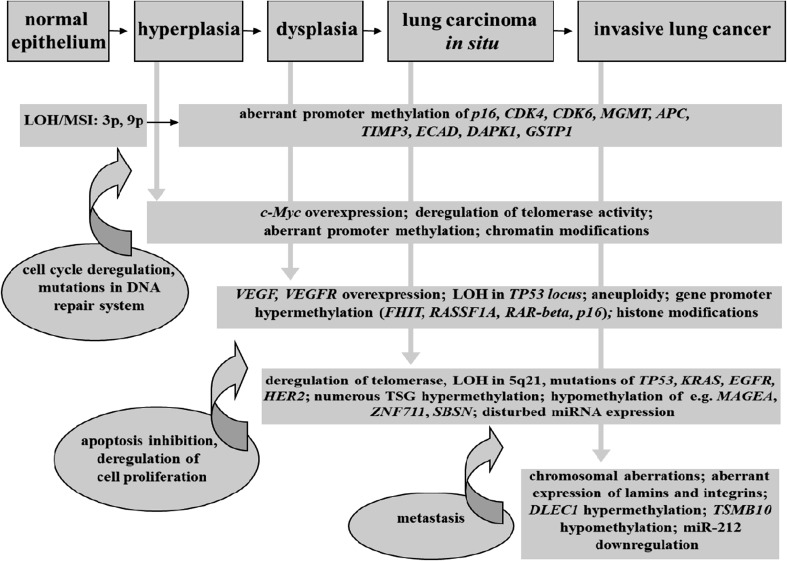
Genetic and epigenetic changes observed in lung carcinogenesis

Although cancer is traditionally regarded as genetic disorder, it has been thoroughly documented that epigenetic changes are very powerful modulators of cellular phenotype in pathological mechanism of carcinogenesis. Figure 1 depicts molecular changes—both genetic and epigenetic—during lung carcinogenesis.

## The effect of epigenetic modifications on gene expression

It is well documented that the presence of methylated cytosines in CpG islands within gene promoter sequence prevents binding of the transcription machinery or allows binding of transcription repressors [8, 10, 14]. So, the effect of DNA methylation is gene silencing related to the inhibition of its transcription. In case of histone modifications, their activating/inhibiting influence on gene transcription depends upon the localization of modified amino acid and specific core histone, the so called “histone code” [15, 16]. Particularly important are the N-terminal modifications of histones H3 and H4. For example, acetylation of lysine 4 in histone H3 (H3K4) enables active gene transcription, and its deacetylation results in gene silencing [15, 17–19]. Regarding methyl group binding to the histones, trimethylation of H3K4 (H3K4me3) is observed in transcriptionally active chromatin, while trimethylation of lysine 27 in H3 (H3K27me3) has suppressive effect [15, 17, 19, 20].

Both DNA methylation and histone modifications are enzymatic processes and are reversible. The enzymes participating in histone modifications are histone acetyltransferases (HATs), histone methyltransferases (HMTs), histone deacetylases (HDACs) and histone demethylases (HDMs) [8, 17, 19]. DNA methylation is mediated by DNA methyltransferases (DNMTs). In human, there are three catalytically active DNMT forms (DNMT1, DNMT3A, DNMT3B). DNMT1 is responsible for maintaining the existing pattern of methylation, while DNMT3A and 3B mediate de novo methylation [12, 21, 22]. Methylated promoter sequence is recognized by methyl binding proteins (MBPs), including MeCP2 (methyl CpG binding protein 2), MBD1 (methyl-CpG binding domain protein 1) and ZBTB 33 (zinc finger and BTB domain containing protein 33; KAISO). These proteins form complexes with transcriptional co-repressors (CRs), as well as HDACs and HMTs [4, 15, 23, 24]. This indicates that these epigenetic processes—DNA methylation and histone modifications—are tightly interrelated, and co-operate in gene expression regulation [1, 12, 15, 18, 20, 25–27]. There are evidences of direct influence of DNA methylation on histone modifications, as well as the effect of histone modifications on de novo DNA methylation [8, 11, 15, 28]. Still, the open question remains which of them occurs first. The results of many research works suggest the opposite findings [26, 28].

Additionally, very recently, it has been discovered that not only protein-coding genes but also micro-RNAs, possessing growth inhibitory function, could be epigenetically silenced in cancer cells [29]. According to the results of analysis of genomic sequences of miRNA genes, approximately half of miRNAs are associated with CpG islands [30], suggesting that the epigenetic events can influence also miRNA expression. This is also confirmed by the findings, that some miRNAs are upregulated when (i) the cells are exposed to the demethylating agents, such as 5-aza-2′-deoxycytidine, (ii) when mutation of DNMT is present or (iii) upon histone deacetylase inhibitor treatment [29, 31, 32]. Epigenetic silencing of miRNAs results in down- or up-regulation of their targets, i.e., tumor suppressor genes or oncogenes of known function in carcinogenesis.

## Altered DNA methylation pattern in carcinogenesis

More than 20 years ago it was found that DNA methylation patterns in cancer cells—in comparison to normal cells—are altered [4, 23, 33]. Methylation analysis of more than 1000 non-selected CpG islands in various primary tumors (e.g., breast, colon, testis, head and neck carcinoma) confirmed disturbed methylation patterns [34]. These alterations involve global genome hypomethylation concerning normally methylated sequences and/or hypermethylation of CpG reach sequences (CpG islands) in promoters of tumor suppressor genes (TSGs) which remain unmethylated under normal conditions [1, 5, 6, 23, 35, 36].

DNA hypomethylation leads to carcinogenesis via three molecular mechanisms: (1) microsatellite instability through activation of repetitive elements (SINE or LINE) or retrotransposons (2) transcriptional activation and overexpression of oncogenes (3) loss of imprinting (LOI) [4, 6, 37, 38].

Global DNA hypomethylation is characteristic for neoplastic transformation from benign to malignant tumors and is known to be increased with cancer progression [37, 39]. On the other hand, regional hypermethylation of TSGs is observed in many human types of cancer, including lung, and plays important role in carcinogenesis [1, 15, 39–46]. These processes—hypo- and hypermethylation—are often observed in parallel in cancer cell, however they are recognized as independent epigenetic changes and are linked with different DNA sequences [23, 35, 37].

TSGs regulate many physiological processes in cell, such as growth, proliferation and apoptosis. They also code for transcriptional factors, adhesion molecules, proteins which regulate angiogenesis as well as participate in DNA repairing system, cell cycle regulation including cyclin-dependent kinase inhibitors and GTP-ase activators [1, 8, 26]. Epigenetic alteration of TSGs involved in regulation of cell cycle may disturb the main cell signaling pathways responsible for regulation of proliferation and apoptosis, while promoter hypermethylation of DNA repair genes prevents DNA repair process and results in accumulation of genetic instability in transforming cell, initiating carcinogenesis [7, 8].

Epigenetic silencing of TSGs is an early event in carcinogenesis. The results of many studies indicate that it is observed also in benign tumors, as well as during early neoplastic progression [1, 23, 36, 40, 47–56]. On the other hand, the aberrant DNA methylation pattern significantly correlates with poorer tumor differentiation, higher malignancy and worse prognosis in a patient. It clearly indicates that DNA methylation increases during cancer progression [40, 47–49, 53, 54, 56].

The proposed model of CIMP (CpG island methylator phenotype) explains cancer development through the simultaneous inactivation of multiple genes, both TSGs and DNA repair genes, as well as including MSI events. The authors of the hypothesis suggested that it could be true for many types of cancer [57]. Indeed, the recent studies, concerning among others stomach cancer, breast cancer and non-small cell lung carcinoma, have indicated CIMP as independent prognostic factor [58–60].

## Altered histone modifications in carcinogenesis

Nucleosomal histones are targets of a large number of post-translational modifications, including methylation, acetylation, phosphorylation, ubiquitination, glycosylation, ADP-ribosylation and sumoylation (see Table 1), among which acetylation has been the most extensively investigated [61], confirming the links to cancer initiation and progression. This modification is dynamically preserved in vivo by the opposing histone acetyltransferase (HAT) and deacetylase (HDAC) actions [62].

Unstructured N-terminal tails (20–30 residues) of core histones (H2A, H2B, H3, H4) contain multiple lysine residues for covalent modifications, including acetylation. Each core histone contains different variants and acetylation occurs on many of them. However, it has been recognized that histone acetylation does not act alone but actively *cross*-*talks* with other histone modifications, as well as with DNA methylation [63]. Histone acetylation and DNA methylation are known to be intimately linked, as mediated by a group of proteins with methyl-binding activity (the above mentioned KAISO, MBD1 and MeCP2) which—after binding to the methylated gene promoters—recruit protein complexes that contain HDACs thereby leading to gene silencing and chromatin condensation.

In normal cells, the non-disturbed histone acetylation pattern (belonging to the above mentioned histone code) controls many cellular and developmental processes, including gene expression, DNA replication, DNA repair or DNA recombination [17]. Thus, the abnormal histone acetylation leads to cancer development via affecting many nuclear and cellular processes [17, 64]. Moreover, disruption of enzyme activities, i.e., HAT or HDACs, may play a significant role in uncontrolled growth and proliferation of tumor cells. The study results have shown that treatment with HDAC inhibitor (trichostatin A, TSA) via increasing histone acetylation results in increased expression of many genes encoding suppressors of invasion and metastasis, negative cell-cycle regulators and apoptosis-related proteins [65].

In addition to changes in histone acetylation, cancer cells are also distinguished by widespread changes in histone methylation patterns. The polycomb repressor complex 2 (PRC2)—which is recognized as a significant functional component for polycomb group gene family—is involved in the initiation of silencing and contains histone methyltransferases that can methylate histone H3 lysine 9 and 27, which are marks of silenced chromatin. The polycomb gene BMI1, a component of PRC1, is overexpressed in several human cancers so that it might be expected that aberrations in this system would give rise to global alterations in gene silencing in cancer [66]. The results of experimental studies indicate the increased level of repressive polycomb mark trimethylated H3K27 in cancer cells relative to a normal cells, resulting in histone modification and target gene silencing [67].

The rapidly emerging data strongly indicate that the entire epigenome is fundamentally disturbed in cancer development. Moreover, these genome-wide changes in the structure of the epigenome can lead to the genomic instability, that is a hallmark of cancer [68]. Accumulating evidence clearly indicates fundamental association between global histone modification levels and tumor aggressiveness, regardless of cancer type [69].

## Epigenetically altered micro-RNA regulation in carcinogenesis

Micro-RNAs are small non-coding RNAs of ~22 nucleotides length. Based on the degree of homology to their 3′UTR target sequence, miRNAs can induce translational repression or degradation of target mRNAs. Although, it has been also described that miRNAs can also increase the expression of mRNA [70]. In human genome, it is estimated that 1,000 miRNAs are transcribed and 30 % of all genes are under miRNA regulation, so one miRNA modulates hundreds of downstream genes via post-transcriptional process. Thus, miRNAs control a wide range of biological processes, including cell development, proliferation and differentiation, as well as apoptosis [71].

As miRNAs play significant roles in the normal functioning of the cells, their deregulation would result in disruption of normal cell functions and lead to diseases as well. Indeed, in many types of human cancer the study results indicate aberrant miRNAs expression—both overexpression and downregulation [72, 73]. Thus, and according to the target mRNAs, miRNAs have been proposed to function as either tumor suppressors or oncogenes.

Similarly like in case of protein-coding genes associated with cancer, the most convincing evidence linking miRNAs to tumorigenesis comes from genetic alterations in cancer cells. Genetic mechanisms leading to the deletion, amplification, or translocation of miRNAs are usually chromosomal abnormalities. Especially, approximately 50 % of human miRNA genes are located at fragile sites or areas of the genome which are prone to breakage and rearrangement in cancer cells. The examples are two tumor-suppressor microRNAs, miR-15a and miR-16-1, that are down-regulated in 70 % of chronic lymphocytic leukemia (CLL) cases due to chromosomal deletions or mutations at their loci [74]. Also, factors involved in miRNA biosynthesis machinery are dysregulated in human tumors [75].

The results of several studies revealed that miRNAs are also under epigenetic regulation. As mentioned earlier, nearly half of miRNAs are associated with CpG islands, making them prone to epigenetic modifications. It has been proven by the findings that some miRNAs are up-regulated upon the exposure of cells to the agent 5-aza-2′-deoxycytidine, or upon mutation of methyltransferases (DNMTs) [29, 31]. Indeed, several miRNAs have been identified that are silenced by CpG hypermethylation in cancer cells, as compared to normal ones. For example, miR-203 frequently undergoes DNA methylation in T cell lymphoma, but not in normal T lymphocytes [76]. Hypermethylated miRNAs have been discovered in several types of cancer, including breast (especially miR-9-1, but also: mir-124a3, mir-148, mir-152, and mir-663) [31], colorectal (miR-124a, miR-43b, miR-34c) [29, 77], oral (miR-137, miR-193a) [78], bladder and prostate tumors (miR-126) [79] or hepatocellular carcinoma (miR-124, miR-203, miR-375) [80]. Epigenetic regulation of miRNA activity is a very cell- and tumor type-specific. An example is let-7a-3 which has been found to be hypermethylated in breast cancer [31], but hypomethylated in lung cancer [81].

Additionally, in cancer cells, not only DNA methylation status but also chromatin structure around miRNA genes differ between cancer and normal cells, as demonstrated in several studies. In bladder cancer Saito et al. [82] revealed, that DNA demethylation and histone deacetylase inhibition could reverse miR-127 expression in cancer cells. Similarly, in the study performed by Bandres et al. [83], treatment with a DNA methyltransferase inhibitor and a HDAC inhibitor restored expression of 3 of the 5 microRNAs (hsa-miR-9, hsa-miR-129 and hsa-miR-137) in colorectal cancer cell lines.

In summary, emerging evidences suggest that epigenetic changes of miRNA represent another mechanism that plays a role in carcinogenesis, resulting in down- or up regulation of the protein product of the target mRNAs. Especially, having in mind that among miRNA target genes are oncogenes, tumor suppressor genes, multiple cell cycle regulators, including cyclins, cyclin-dependent kinases, Rb-family proteins, and Cip/Kip family of cell-cycle inhibitors, antiapoptotic genes, and key sets of genes involved in invasion and migration, as well as those engaged in angiogenesis [84]. The number of the identified epigenetically altered miRNAs is still growing and their function is being elucidated. Although, in many cases, the prediction of specific targets remains a major bio-informatic challenge and future studies will therefore have to address the identification of target mRNAs and the elucidation of the functional consequences of deregulated miRNAs. Table 2 lists examples of miRNAs that undergo epigenetic modifications in human cancers and their target genes.

**Table 2:** Examples of epigenetically modified miRNAs in human cancers (excluding lung cancer) and their identified target genes

miRNA	Epigenetic modification	Cancer	Target gene/infuence	References
miR-9	DNA hypermethylation; DNA hypermethylation/histone deacetylation	Breast cancer, neuroblastoma; colorectal	*TrkC*/overexpression	[31, 83, 84, 148, 149]
miR-34a	DNA hypermethylation	Neuroblastoma	*E2F3*/overexpression	[150, 151]
miR-34b/c	DNA hypermethylation	Colorectal; pancreatic, renal cell, mammary	*BTG4*/TSG silencing *BCL2*, *MET*, *MYC*, *CDK4*/*6*/oncogene derepression	[77, 148, 150]
miR-124a	DNA hypermethylation	Breast, colorectal, hepatocellular, acute lymphoblastic leukemia	*CDK6*/oncogene derepression	[29, 84, 152]
miR-126	DNA hypermethylation	Bladder, prostate	*SPRED1*, *PIK3R2*/derepression	[153]
miR-127	DNA hypermethylation/histone deacetylation	Bladder	*BCL6*/oncogene derepression	[82]
miR-137	DNA hypermethylation/histone deacetylation	Colorectal oral squamous cell carcinoma	*CDK6*/oncogene derepression	[29, 154]
miR-193a	DNA hypermethylation	Oral squamous cell carcinoma	*E2F6*/oncogene derepression	[154]
miR-200	DNA hypermethylation	Ovarian	*ZEB1*, *ZEB2*/overexpression	[155]
miR-203	DNA hypermethylation	Acute lymphoblastic leukemia	*ABL1*/overexpression	[76]
miR-512-5p	DNA hypermethylation/histone deacetylation	Gastric	*MCL1*/oncogene derepression	[156]
let-7a-3	DNA hypermethylation	Breast, ovarian	*HMGA2*, *KRAS*, *NRAS*, *MYC*, *IGF*-*II*/oncogene derepression	[84, 157, 158]

*ABL1* c-abl oncogene 1, non-receptor tyrosine kinase, *BCL2* B-cell lymphoma 2, *BCL6* B-cell lymphoma 6, *BTG4* B-cell translocation gene 4, *CDK4* cyclin-dependent kinase 4, *CDK6* cyclin-dependent kinase 6, *E2F3*-E2F transcription factor 3, *E2F6*-E2F transcription factor 6, *HMGA2* high mobility group AT-hook 2, *IGF-II* insulin-like growth factor 2, *KRAS*-v-Ki-ras2 Kirsten rat sarcoma viral oncogene homolog, *NRAS* neuroblastoma RAS viral (v-ras) oncogene homolog, *PIK3R2* phosphoinositide-3-kinase, regulatory subunit 2, *MCL1* myeloid cell leukemia sequence 1, *MET* met proto-oncogene (hepatocyte growth factor receptor), *MYC* v-myc myelocytomatosis viral oncogene homolog (avian), *SPRED1*-suppressor of Ras/MAPK activation, *TrkC*-tropomyosin-related kinase C, *TSG* tumor suppressor gene, *ZEB1* zinc finger E-box binding homeobox 1, *ZEB2* zinc finger E-box binding homeobox 2

So far, many reports have revealed abnormal microRNA expression in cancer cells, clearly suggesting that it is not an exception, but the rule, in human cancer. Indeed, an aberrant microRNA profiling (miRNome) has been described in almost all human cancers, and could be applied as useful marker for tumor classification, diagnosis, and prognosis.

## Epigenetics in lung cancer

Lung cancer is the leading cause of cancer death worldwide [85, 86]. Although lung cancer diagnoses in men decreases each year and death rates also decline, the incidence of this type of cancer in women increases by 0.5 % each year. Lung cancer in women, as compared with men, occurs at a slightly younger age, and almost half of lung cancer cases in patients under 50 years old occurs in women, even in those who have never smoked. The reason for this is not completely understood. Although cigarette smoking is the primary cause of the increased incidence of lung cancer in women and men, some other etiologic factors, like exposure to secondhand smoke, asbestos, radon or heavy metals also plays some role [86]. Additionally some genetic factors (involving genes which participate in carcinogen metabolism, cell growth control, DNA repair system) and hormones such estrogen (in women) could directly or indirectly affect cancer growth and might contribute to the development of this type of cancer. Five year survival after lung cancer surgery is approximately 10–70 % depending on the stage of the tumor [87].

Primary lung cancer originates from epithelial cell. Four basic histological types are distinguished, representing approximately 95 % of all lung cancers. These are: Squamous Cell Carcinoma/SCC/(40 %), Adenocarcinoma/AC/(30 %) and Large Cell Cancer/LCC/(10 %) belonging to Non-Small Cell Lung Carcinoma (NSCLC), and Small Cell Carcinoma (SCLC). Squamous Cell Carcinomas are associated with cigarette smoking, adenocarcinomas may be found in patients who have never smoked.

Because of treatment concerns, lung cancers are separated into two groups: Non- Small Cell Lung Carcinoma (NSCLC) which accounts for approximately 80 % of all primary lung cancers and Small Cell Lung Carcinoma (SCLC) which accounts for 20 % [86]. NSCLCs are relatively insensitive to chemotherapy and radiation therapy compared with SCLCs. Patients with resectable lesion may be cured by surgery or surgery followed by chemotherapy. Local control using radiation therapy can be achieved in a large number of patients with unresectable disease, but cure is achieved only in a small number of patients. SCLC differs from other histological types in terms of many biological and clinical characteristics (high rates of proliferation, short doubling time of tumor mass, a prominent tendency to early hematogenous spread, chemo- and radiosensitivity) [86].

Epigenetic changes observed in lung carcinogenesis include the three main aberrations: aberrant DNA methylation pattern (hyper- and hypomethylation), histone modifications and non-coding RNA regulation.

## DNA hypomethylation in lung carcinogenesis

Hypomethylation in lung cancer tissue is manifested by: (i) demethylation of the promoter regions of oncogenes; (ii) reduced global amount of methylcytosines in the genome; (iii) demethylation of repetitive DNA, which under physiological conditions is heavily methylated; (iv) increased transcription of the mobile elements of the genome (transposons)—e.g., LINE sequences—and the associated increase in mitotic recombination leading to deletions and translocations.

Aberrant methylation pattern, mainly global hypomethylation of CpG regions, is a hallmark of many cancers, including lung cancer [88]. Global demethylation generally occurs during initiation and progression of cancerogenesis, but in many cases it does not depend on the level of tumor development. Additionally, the results of some studies indicate the significance of not the total demethylation level of the genome but rather of where the hypomethylation is focused in the genome and what genes are affected [89].

So far only a few studies have analyzed global hypomethylation process in primary cancers with the aim of exploring its clinical importance as a molecular marker [90–92].

Long interspersed element (LINE), an abundant class of retrotransposons, occupying nearly 17 % of the human genome, provides a surrogate marker for global hypomethylation [93, 94]. Hypomethylation within the promoter region of potent *LINE*-*1* sequence causes transcriptional activation of *LINE*-*1*, resulting in transposition of the retro-element and chromosomal alteration. *LINE*-*1* methylation status may therefore be a key factor linking global hypomethylation with genomic instability which can lead to the progression of cancerogenesis [95–97]. The association between *LINE*-*1* methylation and genomic instability also suggests that it may be a good prognostic marker in cancer, as previous studies have reported associations between genomic instability and the outcome of cancer patients [97].

Regarding lung carcinoma, *LINE*-*1* hypomethylation was found as an independent marker of poor prognosis in stage IA NSCLC. The results revealed that tumor *LINE*-*1* methylation status may help to select early-stage NSCLC patients requiring adjuvant treatment after curative surgery [98].

Recently, Daskalos et al. [99] have examined the protein related to the p53 tumor protein—TP73 or p73. This protein is involved in cellular stress response and development, cell cycle regulation, and induction of apoptosis. It possesses an intrinsic P2 promoter, controlling the expression of the pro-apoptotic TAp73 isoform and the anti-apoptotic ΔΝp73 isoform. In the studied primary NSCLC samples, the researchers demonstrated the P2 hypomethylation of p73 protein and the associated over-expression of ΔΝp73 mRNA. They found it as a frequent event, particularly in SCCs. Interestingly, P2 hypomethylation strongly correlated with *LINE*-*1* element hypomethylation, indicating that ΔΝp73 over-expression might be a passive consequence of global DNA hypomethylation.

In NSCLC samples the coordinated promoter demethylation of cancer/testis antigens (CTAs)—the potential, novel candidate proto-oncogenes—associated with their upregulation was also found [100]. These genes are expressed in germline cells and in many tumors, but not in normal somatic tissue, apart from testis and placenta. As the study results indicate, CTA expression is tightly associated with a squamous cell histology, and not with lung adenocarcinoma. The coordinated regulation of CTAs and related target genes, like *MAGEA*, *SBSN*, *TKTL*-*1*, *ZNF711*, *G6PD*, has been found to have epigenetic background, correlated with demethylation process. So far, *SBSN, ZNF*-*711* and *G6PD* have not been associated with tumor specific expression or carcinogenesis.

*TKTL1*, encoding a transketolase-like enzyme, has been found recently as a new and independent predictor of survival in patients with NSCLC. Since inhibition of transketolase enzyme activity has recently been shown to effectively suppress tumor growth, *TKTL1* may represent a novel pharmacodiagnostic marker [101].

Another member of the cancer/testis antigen family, *BORIS* (brother of the regulator of the imprinted site, alternative symbols *CTCFL;* CCCTC-binding factor-like protein) is an 11 zinc finger (ZF) protein, which is considered to be a new oncogene. *BORIS* presents a uniquely paired set of genes which dysfunctions may contribute to development of multiple tumor types via epigenetic reprogramming at CTCF target sites, influencing many cell proliferation-associated genes. It has been documented that human *BORIS* is not only aberrantly activated in many types of human cancers, but also maps to the cancer-associated amplification region at 20q13 [102]. Normally, this gene is expressed only in germ cells, but is also aberrantly activated in numerous cancers [102, 103]. The expression of *BORIS* gene is controlled predominantly by changes in DNA-methylation, as its activation requires promoter demethylation. In many types of human cancer, high expression of BORIS protein correlates with the tumor size and grade. Silencing of *BORIS* induces apoptosis in tumorous cell lines [104]. In lung carcinoma cell lines, expression of this gene varies from high to moderate levels [104, 105]. This, in turn, is correlated with the level of *BORIS* promoter methylation [104]. Additionally, *BORIS* positively regulates some of the CTAs by binding and inducing a shift to a more open chromatin conformation with promoter hypomethylation of *MAGEA3*, or independently of promoter hypomethylation in case of *MAGEA2* and *MAGEA4* and thus may be a key effector involved in their de-repression in lung cancer [106]. Additionally, in lung cancer cells the activity of CCCTC-binding factor (CTCF), which influences *Rb2/p130* gene expression is decreased by BORIS, therefore may correlate with progression of lung tumors [107].

The 14-3-3 proteins form a set of seven highly conserved proteins that have recently been implicated in human tumorigenesis. One isoform of the 14-3-3 family, 14-3-3sigma (14-3-3σ), plays a crucial role in G2 checkpoint by sequestering Cdc2-cyclinB1 in the cytoplasm. Radhakrishnan et al. [108] found that the expression level of 14-3-3σ was elevated in the majority of the studied human NSCLC tissues. The gene was hypomethylated in lung tumors as compared with normal lung tissue. This suggests that decreased DNA methylation results in increased expression of 14-3-3σ in NSCLC. There are findings that chemotherapy resistance in NSCLC may also be increased with increased expression of 14-3-3σ via interaction with IGF-1. It has been documented that IGF-1R inhibitors may increase the efficacy of chemotherapy, particularly in SCC [109].

Another observation concerns thymosin β(10) (*TMSB10*). It is a monomeric sequestering protein that regulates actin cytoskeleton organization. *TMSB10* hypomethylation, leading to its overexpression, is a quite frequent event in NSCLC, but probably it is not a common mechanism underlying the gene overexpression [88]. In lung cancer, overexpression of *TMSB10* was correlated with several unfavorable clinicopathological characteristics. What is more, thymosin β(10) is thought to induce microvascular and lymphatic vessel formation by up-regulating vascular endothelial growth factor and vascular endothelial growth factor-C in lung cancer tissues, thus promoting the distant and lymph node metastases and being implicated in the progression of NSCLC [110].

The findings regarding hypomethylation in the above-mentioned genes and their feasibility as diagnostic and prognostic marker are summarized in Table 3.

**Table 3:** Examples of the most frequently hypomethylated genes in lung cancer and their feasibility as clinical markers

Gene	Locus	Clinicopathological effect, clinical marker value	References
*TP73*	1p36.3	Association with SCC; correlation with LINE-1 element hypomethylation	[99]
*MAGEA*: *MAGEA12*, *5*, *4*, *3*	Xq28	Tight association with SCC; correlation with selective growth advantage	[100, 159]
*ZNF711*	Xq21.1	Tight association with SCC; correlation with selective growth advantage	[100]
*G6PD*	Xq28		
*SBSN*	19q13.13		
*TKTL*-*1*	Xq28	Association with shorter patient survival; poor clinical outcome in lymph node negative NSCLCs	[100, 101]
*BORIS*	20q13.31	Regulation of tumor growth and apoptosis, direct and/or indirect (by influencing *Rb2/p130* gene expression) correlation with tumor progression	[104, 105, 107]
*14*-*3*-*3σ*	1p36.11	Increased expression of 14-3-3σ via interaction with IGF-1, particularly in SCC; correlation with increased chemiotherapy resistance	[108, 109]
*TMSB10*	2p11.2	Association with clinical stage, distant metastases, lymph node metastases, poor degree of differentiation, short postoperative survival	[88, 110]

*14-3-3σ* epithelial cell marker protein 1, *BORIS* brother of the regulator of the imprinted site, *G6PD* glucose-6-phosphate dehydrogenase, *MAGEA* melanoma antigen family A, *SBSN* suprabasin, *TKTL-1* transketolase-like 1, *TMSB10* thymosin β 10, *TP73* tumor protein p73, p73, *ZNF711* zinc finger protein 711

## DNA hypermethylation in lung carcinogenesis

As mentioned earlier, hypermethylation of gene promoters is often associated with transcriptional silencing of tumor suppressors. It is believed that this process leads to the initiation and progression of carcinogenesis [111]. Numerous studies have suggested possible clinical usefulness of promoter hypermethylation as marker of early diagnosis and as predictor of patient outcome in lung cancer. There are several studies suggesting various marker panels for lung cancer identification and differentiation.

Kwon et al. [112] have proposed six genes (*CCDC37*, *CYTL1*, *CDO1*, *SLIT2*, *LMO3*, and *SERPINB5*) for lung squamous cell carcinoma (SCC) identification. Especially, methylation pattern of *CYTL1* promoter region was significantly different between early and advanced stages of SCCs. Shames et al. [44] examined seven potential lung cancer markers (*ALDH1A3*, *BNC1*, *CCNA1*, *CTSZ*, *LOX*, *MSX1* and *NRCAM*), three of which showed frequent tumor-specific hypermethylation as compared with normal tissue. In the study performed by Cortese et al. [113] four genes (*FGFR3*, *LAPTM5*, *MDK*, *MEOX2*) were identified as aberrantly methylated in lung cancer. *MEOX2* was uniformly higher methylated in all lung cancer samples, while the methylation of the other three genes was correlated with either the differentiation status of the tumor (*MDK*, *LAPTM5*) or with the tumor histopathological type (*FGFR3*). Another panel of markers for diagnostic application in lung cancer, elucidated recently by Begum et al. [114], consists of six most promising genes (*APC*, *CDH1*, *MGMT*, *DCC*, *RASSF1A*, and *AIM1*). In Chinese population, the results of the study performed by Zhang et al. [115] showed that nine genes (*APC*, *CDH13*, *KLK10*, *DLEC1*, *RASSF1A*, *EFEMP1*, *SFRP1*, *RARβ* and *p16(INK4A)*) had a significantly higher frequency of methylation in NSCLC as compared with normal tissues, while several others (*RUNX3*, *hMLH1*, *DAPK*, *BRCA1*, *p14(ARF)*, *MGMT*, *NORE1A*, *FHIT*, *CMTM3*, *LSAMP* and *OPCML*) showed relatively low sensitivity or specificity. Moreover, the nine genes validated in tumor tissues also showed a significantly higher frequency of tumor-specific hypermethylation in NSCLC plasma, as compared with cancer-free plasma, and a 5-gene set (*APC*, *RASSF1A*, *CDH13*, *KLK10* and *DLEC1*) achieved a sensitivity of 83.64 % and a specificity of 74.0 % for cancer diagnosis.

As far as individual genes are concerned, there are many studies which provide new information on gene-specific hypermethylation. It has been proved that hypermethylation of particular genes correlates differently with tumor’s type, stage and smoking history. However, the methylation frequencies of the genes examined in NSCLC samples vary according to different study results. For example, in the study performed by Yanagawa et al. [116] the methylation frequency was 26 % for *DAPK*, 34 % for *FHIT*, 26 % for *H*-*cadherin*, 14 % for *MGMT*, 8 % for *p14*, 27 % for *p16*, 38 % for *RAR*-*beta*, 42 % for *RASSF1A*, 25 % for *RUNX3*, and 12 % for *TIMP*-*3.* Hypermethylation of *RASSF1* and *DAPK1* was found to be 100 % specific and together identified 39 % of NSCLC tissues. The results performed by Feng et al. [117] revealed high levels of methylation of six genes (*RASSF1, DAPK1, BVES, CDH13, MGMT*, or *KCNH5*) that were 100 % specific and identified 53 % of cancerous tissues. Additionally, some other *loci* showing significant differences in DNA methylation levels between tumor and non-tumor lung tissue have been identified, including: *CDKN2A EX2, CDX2, HOXA1, SFPR1,* and *TWIST1* gene [118]. In another study, it has been documented that in analyzed panel of genes (*3*-*OST*-*2, RASSF1A, DcR1, DcR2, P16, DAPK, APC, ECAD, HCAD, SOCS1, SOCS3)* only *3*-*OST*-*2* followed by *RASSF1A* showed the highest level of promoter methylation in tumors as compared to controls. Moreover, *3*-*OST*-*2* promoter hypermethylation was associated with advanced tumor stage [119]. According to the numerous studies, the most frequently hypermethylated genes in NSCLC are listed in Table 4. Special attention is drawn to the role of gene hypermethylation in histopathological type differentiation, tumor stage, disease outcome and prognosis.

**Table 4:** Examples of the most frequently hypermethylated genes in lung cancer and their feasibility as clinical markers

Gene	Locus	Clinicopathological effect, clinical marker value	References
*RASSF1A*	3p21.3	Correlation with histological type (adenocarcinoma); association with tumor staging, poor survival rate; earlier recurrence in SCC; informative early diagnostic marker for SCC prediction	[114, 116, 119, 120, 160, 161]
*APC*	5q21-q22	Correlation with NSCLC (especially with AC), and shorter survival time	[114, 126, 161, 162]
*FHIT*	3p14.2	Association with a higher susceptibility to lung cancer development; prognostic value in early stage of NSCLC; correlation with AC; marker of disease progression; significant correlation with lymph node metastasis	[116, 127, 161, 163, 164]
*RAR*-*beta*	3p24	Association with advanced stage of NSCLC; correlation with shorter survival time; correlation with AC (diagnostic value); more frequently mathylated in patients with a smoking history	[126, 161, 165, 166]
*MGMT*	10q26	More frequently methylated in smokers and older patients; more common in SCC in males; association with shorter survival time	[161, 167–169]
*RUNX3*	1p36.11	More frequently methylated in ACs; association with shorter survival time	[116, 161]
*CDH13*	16q23.3	Correlation with AD; association with longer survival time	[126, 161]
*CDKN2A*	9p21.3	More frequently methylated in stage IA AC; association with shorter survival time	[118, 161, 164, 170, 171]
*CDH1 CDH1/TIMP3 CDH1/CDH13*	16q22.1	Association with tumor size (3 cm or greater); correlation with SCC; Association with longer survival time	[114, 120, 161]
*DLEC1 DLEC1/hMLH1*	3p22.2	Correlation with advanced stage and lymph node metastasis; Association with shorter survival time	[172, 173]
*DAPK1*	9q34.1	Association with early-stage NSCLC and with shorter survival time; correlation with SCC	[174, 175]

*APC* adenomatous polyposis coli, *CDH1* cadherin 1, *CDH13* cadherin 13, H-cadherin, *CDKN2A* cyclin-dependent kinase inhibitor 2A, p16(INK4), *DAPK1* death-associated protein kinase 1, *DLEC1* deleted in lung and esophageal cancer 1, *FHIT* fragile histidine triad gene, *hMLH1* mutL homolog 1, colon cancer, nonpolyposis type 2, *MGMT* O-6-methylguanine-DNA methyltransferase, *RAR-beta* retinoic acid receptor beta, *RASSF1A* Ras association (RalGDS/AF-6) domain family member 1, *RUNX3* RUNT-related-transcription factor 3, *TIMP3* tissue inhibitor or metalloproteinase

Besides the genes listed in Table 4, the results of studies assign several other hypermethylated genes to the particular histological types of NSCLC. For instance, *CDKN2A, CDX2, HOXA1* and *OPCML* are more frequently silenced via hypermethylation in AC [118], while hypermethylated *CALCA*, *EVX2*, *GDNF*, *MTHFR*, *OPCML*, *TNFRSF25*, *TCF21*, *PAX8*, *PTPRN2*, *PITX2*, reveal correlation with SSC [120, 121].

Additionally, association between gene methylation and patient smoking history has been observed in many studies. Although, some results are opposite. While *CDKN2A*, *DAPK1* and *APC* are found hypermethylated both in smoking and non-smoking lung cancer patients [122–125] others, like *APC, FHIT, RASSF1A* or *CCND2* are strictly correlated with smoking [117, 123, 126–128].

The list of aberrantly methylated genes in lung cancer still expands. In future it might be useful in a more exact prognosis of lung cancer development at early stages of the disease.

## Histone modifications in lung carcinogenesis

Dynamic histone modifications—in cooperation with the above discussed gene promoter methylation—play critical role in the modulation of chromatin conformation and the regulation of gene expression.

In cancer cells, including lung cancer, hypermethylation of promoter CpG islands of thus transcriptionally repressed tumor suppressor genes is associated with a particular combination of histone marks, such as deacetylation of histones H3 and H4, loss of histone H3 lysine 4 (H3K4) trimethylation, and gain of H3K9 and H3K27 trimethylation [1, 8, 15]. Histone H2 and H3 acetylation and trimethylation status in NSCLC and SCLC allows the detection of subpopulations with differential prognosis. This suggests that epigenetic changes associated with histone code play important role in lung cancer tumorigenesis. Excessive acetylation of H4K5/H4K8 and loss of trimethylation of H4K20 was found in NSCLC and pre-invasive bronchial dysplastic lesions. The prognostic value of epigenetic changes involving multiple histones, in particular H2A (H2AK5ac) and H3 (H3K4me2, H3K9ac), is greater in early NSCLC, and the evaluation of these changes may help in selecting early-stage NSCLC patients for adjuvant treatment [129].

The further studies revealed that H4K20 loss of trimethylation identified a subpopulation of early stage (I) lung adenocarcinoma with shorter survival time [130]. Similarly, cellular levels of H3K4me2 and H3K18ac were lower and the observed histone modification patterns were independent predictors of clinical outcome in AC [131]. These results strongly indicated that the lower cellular levels of histone modifications were associated with decreased survival time. Interestingly, the levels of histone modifications correlated positively with each other: loss of one histone modification was generally associated with loss of other modifications within a patient [131].

Additionally, DNA repetitive elements—that are demethylated in cancer DNA—may also get demethylated and/or deacetylated on their associated histones. The biological effects of these alterations of histones at repetitive elements are unclear but the researchers suggest that they probably are associated with a more aggressive phenotype [131]. Generally, increased frequency of cancer cells with lower global levels of histone modifications indicates poorer clinical outcome, i.e., increased risk of tumor recurrence and/or decreased survival time. It has been demonstrated for several cancers, including lung cancer [69].

The mechanisms affecting histone modifications are still being discovered, and could be attributed to improper targeting, altered expression and/or activity of histone-modifying enzymes because of genetic mutations, expression changes, and/or posttranslational control [132]. The aberrant transcription of genes that encode histone acetyltransferase (HAT) or histone deacetylase (HDAC) enzymes or their binding partners, has been clearly linked to carcinogenesis [133]. The study results revealed that stronger HDAC1 expression in tumor cells was an independent predictor of a poor prognosis in patients with adenocarcinoma of the lung [134]. Thus, histone deacetylase inhibitors (HDACis) are now attracting attention as promising therapeutic agents for the treatment of cancer. The effects of HDAC inhibitors on gene expression are highly selective, leading to transcriptional activation of certain genes such as cyclin-dependent kinase inhibitor p21WAF1/CIP1 and repression of others. HDAC inhibition not only results in acetylation of histones but also transcription factors, such as p53, GATA-1 and estrogen receptor-alpha [135]. The results of numerous studies indicate that inhibition of HDACs by HDACis leads to transcriptional activation of genes involved in cancer cell growth, apoptosis, differentiation, migration and invasion. As demonstrated in several studies, induced acetylation of histones H3 and H4 by TSA in lung cancer cells led to re-expression of a number of TSGs, including *TGFBR2*, *SATB1*, *C/EBPalpha, MYO18B*, *DAPK* [67, 136–140]. The comprehensive study performed by Zhong et al. [141], involving high-throughput gene expression microarrays combined with pharmacologic inhibition of DNA methylation and histone deacetylation in NSCLC, uncovered over 200 genes upregulated by 5-azadC and TSA treatment. Some of those genes were induced by TSA alone (*NRIP3*, *CYLD*, *CD9*, *ATF3*, *OXTR*), confirming the role of histone deacetylation in their silencing. Table 5 summarizes the examples of genes and sequences targeted by histone modifications in lung cancer.

## Epigenetic regulation of microRNA in lung carcinogenesis

So far, deregulation of miRNA expression has been demonstrated in lung cancer and its diagnostic, prognostic and therapeutic significance has been underlined. The number of aberrantly expressed miRNAs is still growing. Additionally, it has been observed that global decrease of miRNA expression causally contributes to the transformed phenotype during lung tumorigenesis [142].

As mentioned earlier, besides genetic mechanisms disturbing miRNA expression, also epigenetic events can modulate the expression and regulatory role of miRNA. Aberrant regulation at this level was confirmed also in lung cancer. For instance, the expression of miRNA-124a was found to be epigenetically silenced by DNA hypermethylation and, as the study results have shown, DNA methyltransferase inhibitor (DNMTi) restored its expression [29]. Conversely, micro RNA let-7a-3 is upregulated in lung adenocarcinoma via hypomethylation. Its expression is associated with enhanced tumor phenotypes and oncogenic changes in transcription profiles [81].

Expression of miR-34 family—involved in lung carcinogenesis through p53 pathway—has also been reported to be regulated by DNA methylation. The results of recent analysis have revealed that promoter hypermethylation of miR-34b/c was correlated with a high probability of recurrence and associated with poor overall survival and disease-free survival in stage I NSCLC [143]. This was a relatively common event in NSCLC and might be regarded as a potential prognostic factor for stage I NSCLC.

Regarding another micro RNA with pro-apoptotic function and strongly downregulated in lung cancer, namely miR-212, Incoronato et al. [144] have found that its transcriptional inactivation in lung cancer is not associated to DNA hypermethylation status but to a change in the methylation status of histone tails linked to the promoter region of this microRNA. As the study results have shown, transcriptional silencing of miR-212 in NSCLC involved H3K27me3/H3K9Ac or H3K9me3/H3K9Ac-associated histone modification. Additionally, miR-212 downregulation was tightly associated with the severity of the disease, being significantly suppressed in T3/T4 staging rather than in T1/T2 staging [144].

Another mechanism underlying the regulatory role of micro-RNA and associated with epigenetic modifications has been discovered in case of microRNA-449. This miRNA belongs to the group of so called epi-miRNAs, interacting with the members of epigenetic machinery. The study results indicate that downexpression of miR-449a/b might be one of mechanisms responsible for over-expression of HDAC1 in lung cancer. MicroRNA-449a/b, possessing tumor suppressor function, inhibits cell growth and anchorage-independent growth. It has been shown, that co-treatment with miR-449a and HDAC inhibitors leads to significant tumor growth reduction as compared with HDAC inhibitor mono-treatment. These results suggest that miR-449a/b might be a potential therapeutic candidate in patients with primary lung cancer [145]. Another epi-miRNAs belong to miR-29 family (29a, 29b, and 29c), which target the de novo DNA methyltransferases DNMT-3A and 3B. In lung cancer miR-29s expression has been found to be inversely correlated with both enzymes [146]. According to the study results, the enforced expression of miR-29s in lung cancer cell lines results in a global reduction of DNA methylation, thus leading to re-expression of methylation-silenced tumor suppressor genes (*FHIT*, *WWOX*) and, additionally, inhibits tumorigenicity in vitro and in vivo.

The study findings support a role of epi-miRNAs in epigenetic normalization of NSCLC. They could be a basis for the development of miRNA-based strategies for the treatment of lung cancer (Table 5).

**Table 5:** List of genes and sequences deregulated by histone modifications in lung cancer

Histone modifications	Target gene/sequence	References
Histone deacetylation at target gene promoters	*IL*-*20* and its receptors (*IL*-*20RA/B* and *IL*-*22R1*)	[176]
H3-Ac(-)/H3K4-Me(+/−)/DNA-Me(-)	*TGFBR2*	[136, 137]
Loss of H3K9ac	*SATB1*	[67]
Histone deacetylation at target gene promoters	E prostanoid (EP) receptors 2–4	[177]
Loss of H3K18ac	Repetitive DNA elements: D4Z4, Sat2	[131]
Loss of H3K4me2	Repetitive DNA elements: D4Z4, NBL2	[131]
H3 deacetylation at target gene promoter	*VILIP*-*1*	[178]
Histone deacetylation at target gene promoters	*NRIP3*, *CYLD*, *CD9*, *ATF3*, *OXTR*	[141]
H3 and H4 deacetylation at target gene promoter	*C/EBPalpha*	[138]
H3 and H4 deacetylation at target gene promoter	*MYO18B*	[139]
Histone deacetylation at target gene promoter	*DAPK*	[140]

*ATF3* activating transcription factor 3,* CD9* CD9 antigen,* C/EBPalpha* tumor suppressor CCAAT/enhancer-binding protein-alpha,* CYLD* cylindromatosis,* DAPK* death-associated protein kinase,* IL-20* interleukin 20,* MYO18B *myosin XVIIIB,* NRIP3* nuclear receptor interacting protein 3,* OXTR* oxytocin receptor,* SATB1* special AT-rich binding protein 1,* TGFBR2* transforming growth factor, beta receptor II,* VILIP-1* visinin-like protein-1

The findings on miRNA regulation via epigenetic mechanisms in lung cancer are listed in Table 6.

**Table 6:** Epigenetic regulation of miRNAs in lung cancer

Epigenetic regulation		miRNA	References
Direct	miRNA promoter hypermethylation	miR-34b/c, miR-124a	[29, 143]
	miRNA promoter hypomethylation	let-7a-3	[81]
	miRNA histone modification	miR-212	[144]
Indirect	miRNA influence on HDAC1 overexpression	miR-449	[145]
	miRNA influence on DNMT-3A and DNMT-3B overexpression	miR-29a, b, c	[146]

## Conclusions

In the past decade epigenetics has been one of the most promising and rapidly expanding research fields. Since the discovery of altered DNA methylation patterns, i.e., global DNA hypomethylation and tumor suppressor promoter hypermethylation in cancer cells, in 1980s–1990s, a variety of other epigenetic changes have been identified. They include a wide spectrum of histone modifications and the role of non-coding RNAs. Nowadays a huge amount of knowledge confirms that the epigenetic setting is completely disturbed in human cancer. Epigenetic processes play a key role in the onset and progression of numerous types of tumors.

In lung cancer promoter DNA hypermethylation, identified for still growing number of TSGs, and single gene hypomethylation, are recognized as belonging to the earliest events in lung carcinogenesis and increasing with the disease progression. Cancer-specific hypermethylation and hypomethylation events can be recognized in lung tissue biopsies, but also in biological fluids, like sputum, allowing to predict lung cancer incidence using non-invasive methods. Specific epigenetic biomarkers, associated with hyper- or hypomethylation, can distinguish NSCLC subtypes or reveal early stages of lung tumorigenesis affecting smokers. Aberrant methylation of certain genes is associated with poor outcome and can be useful for the establishment of lung tumor prognosis.

In the recent years, histone-based and miRNA-based epigenetic signatures have also been incorporated into the molecular search for biomarkers in cancer. Specific histone code can support the selection of early stage disease patients and predict their clinical outcome. Additionally, the role of miRNAs, the very recently discovered level of epigenetic control, is gaining importance. The aberrant expression of several miRNAs has been associated with differential diagnosis of NSCLC as well as with survival and cancer recurrence in lung cancer patients.

As potentially reversible, epigenetic aberrations are the targets for potential treatment strategies in cancer therapy. The growing number of reports outline the usefulness of the epigenetic events for lung cancer diagnostic, prognostic and targeted epigenetic therapy.
